# Real-Time Analysis of the Stability of Oil-In-Water Pickering Emulsion by Electrochemical Impedance Spectroscopy

**DOI:** 10.3390/molecules25122904

**Published:** 2020-06-24

**Authors:** Qiuyan Jiang, Ning Sun, Parveen Kumar, Qiuhong Li, Bo Liu, Aixiang Li, Weiwei Wang, Zengli Gao

**Affiliations:** 1 School of Materials Science and Engineering, Shandong University of Technology, Zibo 255000, China; joybella5156@163.com (Q.J.); s15550326021@163.com (N.S.); Kumar@sdut.edu.cn (P.K.); axl@sdut.edu.cn (A.L.); wangweiwei@sdut.edu.cn (W.W.); gaozengli@sdut.edu.cn (Z.G.); 2 Lab of Functional Molecules and Materials, School of Physics and Optoelectronic Engineering, Shandong University of Technology, Zibo 255000, China

**Keywords:** electrochemical impedance spectroscopy, stability, Pickering emulsion, ferrocene, resistance

## Abstract

In this paper, electrical impedance spectroscopy (EIS) was applied to investigate the stability of oil-in-water (O/W) Pickering emulsions prepared with negatively charged silica nanoparticles in combination with a trace amount of redox switchable fluorescent molecules, ferrocene azine (FcA). Electrical impedance values of emulsions obtained at different emulsification speeds were estimated according to the frequency response data with frequencies ranging from 1 MHz to 1 Hz. The equivalent circuit model of toluene-in-water emulsion was established by the resistor (R_O/W_) and capacitor (C_O/W_) in parallel connection. Nyquist diagrams for the emulsions prepared by toluene and water were characterized by the formation of one semi-circle. The droplet size distribution is one of the important factors that affect the stability of the emulsion, except for the volume fraction of water and oil, the size of stabilizing particles, etc. The average particle size of the emulsion droplets decreased as the emulsification speed increased, indicating the higher stability of the emulsion. It was found that the fitted impedance value R_O/W_ of the emulsion decreased with decreasing particle size prepared at different emulsification speeds and storage time by performing real-time EIS detection techniques. The results suggested that EIS could be used to characterize the stability of a toluene-in-water emulsion stabilized by FcA modified silica nanoparticles. Moreover, based on the good electrochemical activity of the FcA molecule, the stability of the Pickering emulsion can be modulated by adding oxidant and reductant and detected by EIS in real-time.

## 1. Introduction

An emulsion dispersion system constructed from solid particles as an emulsifier is called a Pickering emulsion, which enhances emulsion stability by adsorbing solid particles on the surface of the droplets to form an irreversible mechanical barrier film to prevent coalescence between the droplets [1,2,3,4]. Pickering emulsion has been widely studied because of its advantages, such as small dosage, low toxicity and environmental friendliness [5,6]. The stability of the emulsion is one of the important criteria for measuring its quality, which affects the storage and performance of the emulsion. The stability of a Pickering emulsion can be affected by many factors, such as surface wettability, concentration of solid particle emulsifier, shape of the solid particles, pH value, and electrolyte [7,8,9,10,11]. The macroscopic expressions of poor emulsion stability are the oil moisture layer, droplet coalescence, flocculation, etc. Since two or more small droplets aggregate into large droplets, the average particle size of the emulsion increases and the surface film of the large droplets gets thinner than the small droplet film, making them prone to get broken and to coalesce [12,13].

The importance of emulsion stability makes stability characterization become a research hotspot. Heuzey et al. proposed an emulsification index method to describe emulsion stability by measuring the excess aqueous phase height of the stabilized emulsion and the height of the emulsion layer as a function of storage time [14]. The results showed that the small emulsification index of the emulsion indicated the long storage time of the emulsion, imparting higher stability. The interfacial tension and rheological behavior of the emulsion are also one of the commonly used methods for the analysis of emulsion stability because of the formation of the interface film on the surface of the droplets and the interfacial properties of the oil-water phase affect the stability of the emulsion. Oscillatory measurements and steady shear tests were used to study the bulk rheology of Pickering emulsion. By analyzing the storage and loss modulus as a function of angular frequency, the viscoelasticity of the emulsion was known to predict the stability of the emulsion [15]. This method is simple to operate and can roughly reflect the macroscopic phenomenon of the emulsion, but it cannot explain the influence of the change in the microscopic composition of the emulsion system on stability. The size and distribution of emulsion droplets are one of the important factors affecting the stability of emulsions. From the perspective of emulsion kinetics, under the same coalescence rate, the emulsion with smaller droplets takes a longer time to delaminate and the emulsion will be more stable. However, the emulsion is a thermodynamically unstable dispersion system. Since the interface area of the oil-water phase for smaller droplets and the interface energy of the system are larger, therefore, the droplets tend to coalesce with each other to reduce the interfacial energy, thereby the emulsion tends to be unstable.

Electrochemical impedance spectroscopy (EIS) uses a sinusoidal electrical signal with a small enough amplitude to perturb the electrode, and the electrode responds with a corresponding sine wave, causing a sinusoidal current, a phase angle, and a change in current relative to the applied potential. The frequency response function is the AC impedance. EIS has become a powerful tool for studying the physicochemical properties and stability of emulsions, for example, it was used to study the molecular aggregation process and to determine the critical micelle concentration [16,17]. Compared to currently used emulsion stability detection technology, EIS requires a small sample volume and can simultaneously detect multiple samples. At the same time, good emulsification and droplet agglomeration demulsification processes can be captured at lower frequencies.

Oil-in-water (O/W) Pickering emulsion was prepared by combining the electrochemically active fluorescent molecule, ferrocene azine (FcA) with negatively charged silica nanoparticles as the solid particle emulsifier, which was explored in previous work [18]. Herein, previous work was extended by measuring the frequency response data of the O/W Pickering emulsion combining with the electrochemical impedance spectroscopy. An equivalent circuit model was established by connecting a resistor (R_O/W_) and a capacitor (C_O/W_) in parallel. The relationship between the size of the emulsion droplets and R_O/W_ was studied during the phase separation of the emulsion. The results showed that EIS is an effective method to characterize the stability of emulsions. In addition, based on the redox activity of FcA, by alternately adding oxidant and reductant, the stability of emulsions changed between stable and unstable at ambient temperature by redox stimuli, and EIS can be used to detect this conversion of the emulsion in real-time.

## 2. Results and Discussion

### 2.1. The Establishment of an Equivalent Circuit 

**Figure 1:**
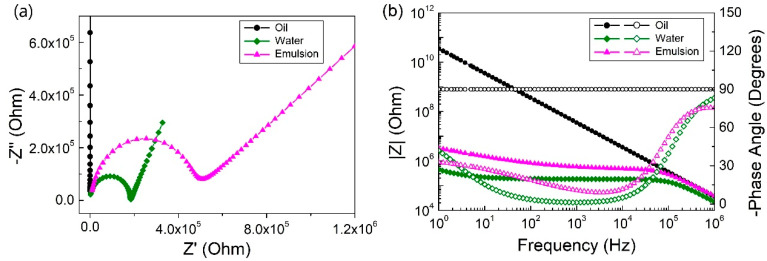
(**a**) Nyquist and (**b**) Bode diagrams for oil, water, and Pickering emulsion. The solid symbols are the impedance modulus-frequency curve and the hollow symbols are the phase angle-frequency curve in the Bode diagram (**b**).

**Figure 2:**
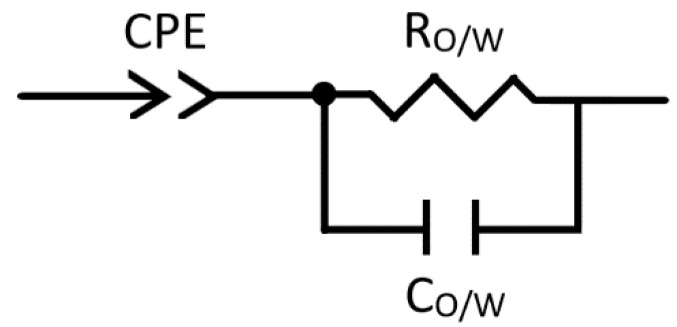
Equivalent circuit of O/W Pickering emulsion.

The Bode and Nyquist diagrams of oil, distilled water, and toluene (9 mL)-in-water (6 mL) Pickering emulsion with droplet size 25.89 ± 8.57 μm are shown in Figure 1. One semicircle of the high frequency part and a straight line with an angle greater than 45 degrees from the real axis at low frequency region could be observed, and the appearance of a semicircle in the Nyquist diagram indicated one time constant and one corresponding state variable in the measured frequency range, primarily describing the electrochemical behavior of the Pickering emulsion. The Bode diagrams (Figure 1b) show that the trend of phase angle and impedance modulus corresponded to the characteristics of the capacitor. It could be observed that the increase in the impedance phase ends with a constant phase at the lowest frequency, which might be caused by electrode interface polarization [19,20]. Therefore, a constant phase element (CPE) was added to the circuit in order to well establish the equivalent circuit [21,22]. Due to the non-conductivity of the organic solvent, no relevant resistance value could be obtained by EIS test for the single toluene phase. Since the O/W Pickering emulsion was an oil-water mixed system, where water acted as a continuous phase to form a current path in the emulsion, which had much more resistance than the pure water single-phase system. The equivalent circuit (Figure 2) for the Pickering emulsion system can be used in parallel with the resistor (R_O/W_) and capacitor (C_O/W_). The electrochemical frequency response impedance data of the emulsion measured and recorded by the electrochemical workstation in the frequency range of 1 MHz–1 Hz was analyzed by Zview software to obtain Table 1. It can be seen from Table 1 that the equivalent circuit analog value agreed well with the experimental value, indicating that this equivalent circuit could be used to characterize the test system.

### 2.2. EIS Characterization of Pickering Emulsion

**Figure 3:**
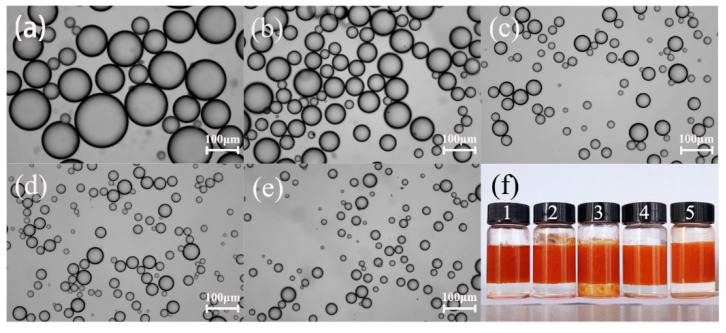
Microscopic images of a toluene (9 mL)-in-water (6 mL) Pickering emulsion prepared at different emulsification speeds (**a**) 4000 r/min, (**b**) 6000 r/min, (**c**) 8000 r/min, (**d**) 10,000 r/min, (**e**) 12,000 r/min with different droplet sizes 87.65 ± 35.52 μm, 49.52 ± 17.88 μm, 29.16 ± 10.93 μm, 25.89 ± 8.57 μm, 17.28 ± 7.75 μm, respectively, and (**f**) visual photograph of the Pickering emulsion.

**Figure 4:**
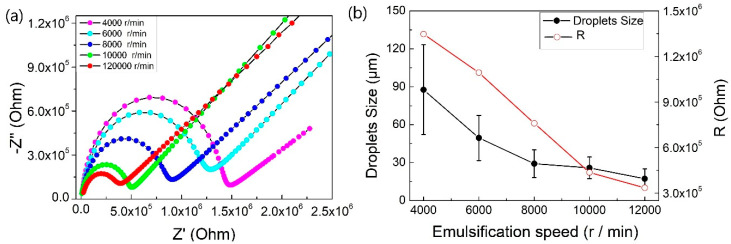
(**a**) Nyquist diagram of Pickering emulsions with different droplet size distributions and (**b**) emulsion droplet size change and corresponding fitting resistance value at different emulsification speeds.

Figure 3 shows the visual photograph of the Pickering emulsion and optical micrographs of the Pickering emulsion droplets prepared at different emulsification speeds. The smaller and more uniform average particle size of the emulsion was obtained by increasing the emulsification speed. The Nyquist diagram (Figure 4a) of emulsions with different average particle sizes displayed that the semi-circle shown in the high frequency part of the Nyquist diagram represented the electrochemical behavior of the Pickering emulsion. Therefore, we focused on this part in the following discussion.

The equivalent circuit fitting values shown in Table 2 indicated that as the droplet size increased, the resistance of the emulsion also increased significantly. The change in emulsion droplet size and corresponding fitting resistance value at different emulsification speeds are shown in Figure 4b. It can be seen that the capacitance value decreased when the relaxation process became longer. The increase in resistance might be due to the increase in droplet size, which resulted in faster coalescence between droplets. Then the larger droplets after coalescence moved up to the surface of the electrode because of the influence of buoyancy and were compressed into a sphere. Therefore, the water passage between the droplets decreased and the migration of charge carriers was hindered to some extent. This behavior can be described by the Bond number [23]:

$$Bo=\frac{net\;gravitational\;force}{interfacial\;forces}=\frac{\Delta \rho \nu g}{2\gamma /R}\approx \frac{\Delta \rho gR^{2}}{\gamma }$$

where *Δρ* is the difference in density between the dispersed phase and the main medium, *V* is the droplet volume, *R* is the droplet radius, and *γ* is the interfacial tension between the two phases. The equation shows that the ratio of buoyancy per unit area to interfacial tension is proportional to *R*^2^. 

The droplets having a large average diameter were easily compressed and joined into one sheet due to the large buoyancy, thereby reducing the current path between the electrodes which could be supplied to the emulsion system with respect to distilled water and small particle size emulsions [24]. It can be concluded from the EIS measurement results that the resistance value was larger for poorly stable emulsions. The decrease of C_O/W_ was mainly attributed to the fact that the dielectric constant of toluene was much smaller than the dielectric constant of pure water. As the particle size of the droplet increased, the volume of the oil phase of the emulsion layer increased and the value of capacitance also approached more towards the oil phase [25].

To further characterize the stability of emulsion with time, we performed continuous EIS observations on the emulsion over three weeks and optical micrographs of the corresponding emulsion droplets were captured, to investigate the relationship between emulsion stability, emulsion particle size distribution, and storage time. Upon visual observation, the emulsion was in a stable state for up to 10 days and the lower aqueous phase was transparent. 

**Figure 5:**
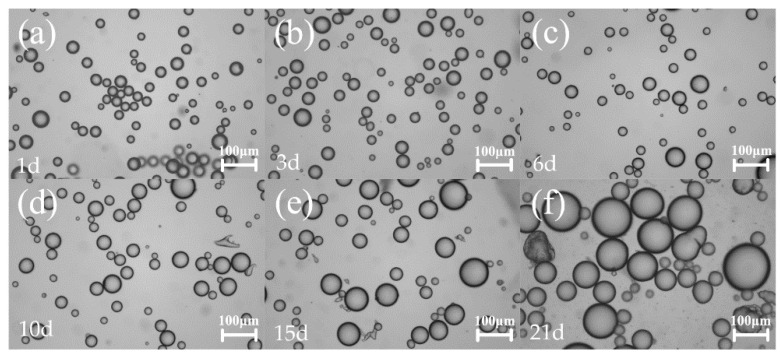
Microscopic images of Pickering emulsions with different droplet sizes at different storage times. The droplet sizes from (**a**) to (**f**) were 25.06 ± 6.27μm, 25.89 ± 7.30 μm , 26.28 ± 7.02 μm, 28.30 ± 8.93 μm, 42.68 ± 15.05 μm, 65.36 ± 22.85 μm.

**Figure 6:**
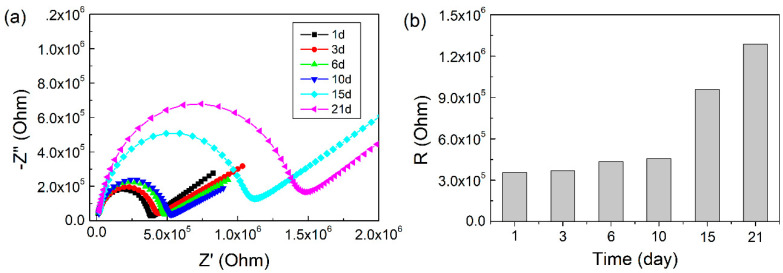
(**a**) Nyquist diagrams of Pickering emulsions with different standing time and (**b**) corresponding fitting resistance values.

Figure 5 shows the optical micrographs of emulsions with different storage times. It can be seen that the size of emulsion droplets did not change after up to six days of storage time, but increased significantly after ten days due to coalescence, which was consistent with the results obtained by the impedance measurement (Figure 6a). No obvious change of radius in the impedance semi-circle at the high-frequency region was observed up to a storage time of ten days. However, due to the continuous detection of the state of the emulsion, the insertion of the electrode caused the emulsion to be unstable. After half a month, solid particle emulsifier fallen off the surface was found at the bottom of the cell. The solid particle film formed by the adsorption of the solid particles at the oil-water interface became thinner, and the droplet-particle or droplet-droplet repulsions were reduced. Under the Ostwald ripening and buoyancy, the increase of droplet size was observed by a microscope. The equivalent circuit fitting electrical values of Pickering emulsions at different storage times are shown in Table 3. When the stability of the emulsion deteriorated, the value of the corresponding R_O/W_ increased. Figure 6b indicates that the value of R_O/W_ increased when the particle size of the emulsion increased. Thus, it could be concluded that the stability of the emulsion is related to the average particle size of the droplets as the emulsion with a large average particle exhibited poor stability and a larger resistance value. 

**Figure 7:**
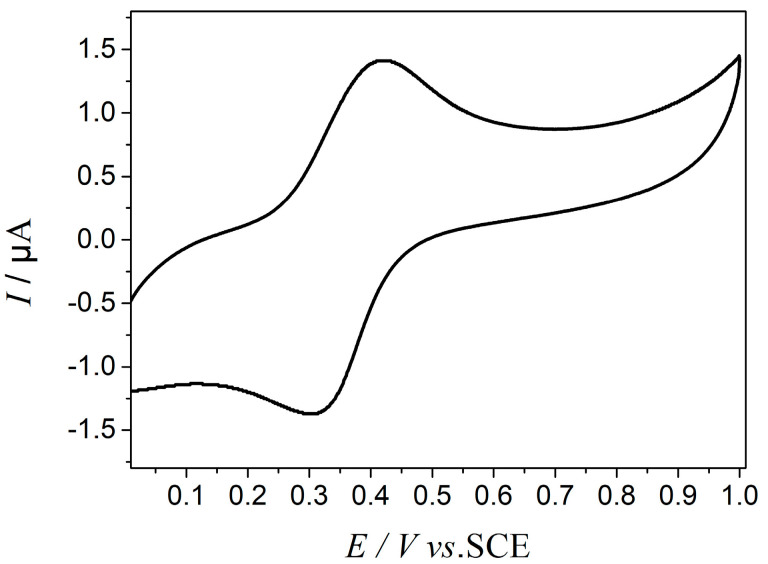
Cyclic voltammogram of FcA in 0.1 mol L^−1^ KCl solution at a scan rate of 50 mV s^−1^.

**Figure 8:**
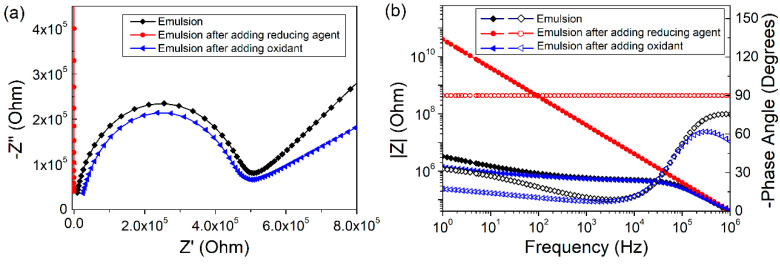
(**a**) Nyquist and (**b**) Bode diagrams before and after the redox regulation of the Pickering emulsion. The solid symbols are the impedance modulus-frequency curve and the hollow symbols are the phase angle-frequency curve in the Bode diagram (**b**).

**Figure 9:**
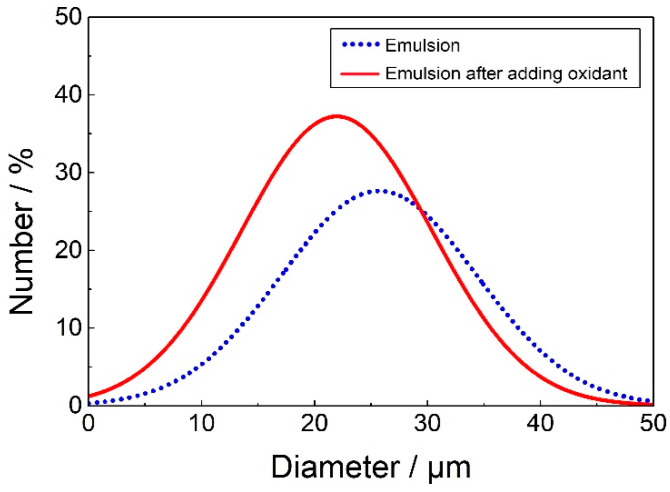
Droplets size distribution of the Pickering emulsion before and after redox regulation.

**Figure 10:**
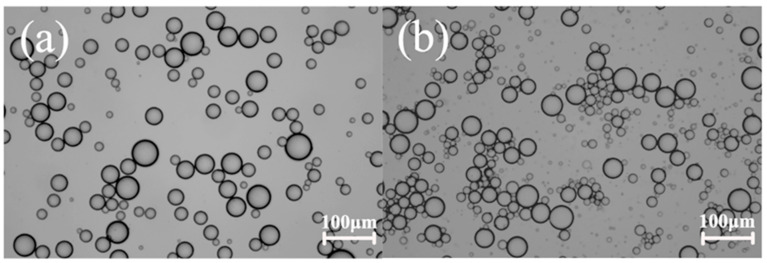
Microscopy images of a Pickering emulsion with droplet size (**a**) 25.43 ± 8.34 μm and (**b**) 21.97 ± 8.39 μm before and after redox regulation.

Figure 7 shows the cyclic voltammogram of FcA obtained by applying a potential scan from 0 to 1 V at 50 mV s^−1^. The obvious redox peak can be observed, indicating the good redox activity of FcA. When a trace amount of reducing agent, hydrazine hydrate was added into the Pickering emulsion, the emulsion started to flocculate and stratify because of the desorption of FcA molecules from the silica surface. Almost no droplets in the upper layer of the emulsion were observed after demulsification, so the current path could not be formed between the oil phases. The EIS measurement indicated that the emulsion system was a pure capacitance response (Figure 8). When a trace amount of hydrogen peroxide was added and emulsified by homogenizing, the emulsion was reformed. The corresponding droplet size distribution was shown in Figure 9. It can be seen that the size is slightly smaller after the emulsion is reformed by adding oxidant (Figure 10), which is consistent with the result of EIS measurement shown in Figure 8.

As predicted, the Nyquist diagram showed that the R_O/W_ for the emulsion reformed was similar to that of R_O/W_ for the preregulated emulsion. Thus, the stability of the emulsion could be modulated by adding oxidant and reductant. On the other hand, EIS technology could be used as a method to detect and expand the emulsion stability pathway. Further work needs to be done to realize the regulation and real-time monitoring of emulsion by the electrochemical method.

## 3. Materials and Methods 

**Figure 11:**
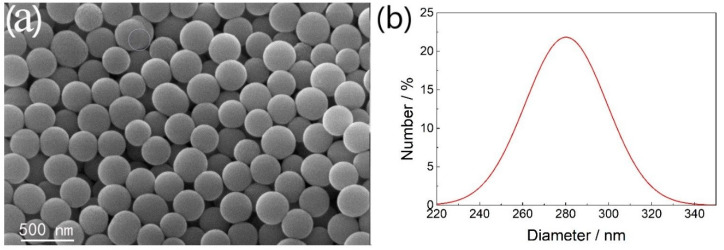
(**a**) SEM images and (**b**) size distribution curve of SiO_2_ particles.

Toluene, hydrazine hydrate, and hydrogen peroxide (80%) were purchased from Sinopharm Chemical Reagent Co., Ltd (Shanghai, China). Triple distilled water was used throughout the experiment. All reagents were used as received without further purification. Oxidized FcA (Fc^+^A) modified SiO_2_ solid particles were prepared according to the procedures reported in the literature [18]. The morphology of SiO_2_ particles can be characterized by SEM, as shown in Figure 11. It could be seen that SiO_2_ nanoparticles with a uniform size were observed and the particles size was about 260 nm.

About 0.3 wt.% of FcA modified SiO_2_ particles was ultrasonically dispersed in distilled water (6 mL) and then toluene (9 mL) was added to the dispersion. The oil/water mixture was then homogenized using FJ200-SH digital display high speed dispersion homogenizer (Shanghai Specimen and Model Factory, Shanghai, China) for 5 min at different speeds. The emulsion type was identified by dye method.

A drop of freshly prepared Pickering emulsion was placed onto the slide and gently covered with a coverslip. Micrographs for emulsion droplets were observed by UB103i biological microscope (Chongqing UOP Photoelectric Technology Co., Ltd. Chongqing, China). The average droplet size of the emulsion was estimated by Nano Measurer 1.2 software.

Electrochemical measurement of the samples was performed on a CHI 660E electrochemical workstation (Shanghai Chenhua Instrument Co., Ltd. Shanghai, China) with a typical cell (volume = 15 mL) attached with three-electrodes: saturated calomel as a reference electrode (SCE), platinum foil as a counter-electrode, and a glassy carbon electrode as a working electrode. Prior to the experiment, the surface of the working electrode was polished using Buehler alumina powder (Lab Testing Technology (Shanghai) Co., Ltd. Shanghai, China) to a mirror finish. The electrode was then rinsed with deionized water and the FcA suspension was coated onto the work electrode and dried naturally in the air. Finally, 0.05 wt.% Nafion solution in ethanol was casted on the surface of the sample. Cyclic voltammetry was obtained by applying a potential scan at 50 mV·s^−1^ from 0 to 1 V. 

EIS measurements were performed by using a two-electrode device with the distance 1 cm between them, platinum foil as a counter-electrode, and a glassy carbon electrode as a working electrode. The auxiliary electrode wire and the reference electrode wire were connected to the platinum electrode. The Pickering emulsion was statically placed in the electrolytic cell, the surface of the working electrode was polished using Buehler alumina powder to a mirror finish. The platinum electrode and glassy carbon electrode were rinsed with deionized water and inserted into the emulsion layer. The electrochemical impedance data of the emulsion were measured and recorded using the electrochemical workstation with the frequency response ranging from 1 MHz to 1 Hz. The raw data of the electrochemical impedance spectrum were analyzed by Zview software to establish a suitable equivalent circuit for the emulsion system. 

## 4. Conclusions

This work explored the stability of O/W Pickering emulsions using EIS measurement. Pickering emulsions were prepared using negatively charged silica nanoparticles in combination with a trace amount of redox switchable fluorescent molecules, FcA. Impedance measurements indicated that the O/W Pickering emulsion was characterized as a semi-circular shape in the Nyquist diagram. An equivalent circuit model was established by parallel resistor (R_O/W_) and capacitor (C_O/W_). The quantification of emulsion stability was provided by fitting the EIS results to the parameters (resistance and capacitance) obtained in the equivalent circuit. The average droplet size affected the emulsion stability as R_O/W_ was directly proportional to the average particle size of the droplets and inversely proportional to the stability of the emulsion. Thus, EIS proved to be a suitable technique for characterizing the stability of the O/W Pickering emulsion made from modified silica particles. Further real-time characterization of emulsion stability could be important for both the storage period of the emulsion and the emulsion-demulsification process, such as transportation and extraction of crude oil.

## Figures and Tables

**Table 1:** Fitted value and error of the parameters of Pickering emulsion equivalent circuit.

Parameter	Fitted Value	Error %
R (MOhm)	0.44	2.19
C (pF)	5.02	2.66
CPE-T(μF)	0.16	4.13
CPE-P	0.42	2.24

**Table 2:** Equivalent circuit fitting electrical value of Pickering emulsions with different droplet sizes.

Speed (r/min)	Average Droplet Size (μm)	R (MOhm)	C (pF)	τ (μs)
4000	87.65 ± 35.52	1.35	3.92	5.28
6000	49.52 ± 17.88	1.09	4.00	4.37
8000	29.16 ± 10.93	0.76	4.02	3.05
10000	25.89 ± 8.57	0.44	5.02	2.18
12000	17.28 ± 7.75	0.33	5.15	1.72

**Table 3:** Equivalent circuit fitting electrical values of Pickering emulsions at different storage times.

Storage Time (Day)	Average Droplet Size (μm)	R (MOhm)	C (pF)	τ (μs)
1	25.06 ± 6.27	0.35	3.91	1.38
3	25.89 ± 7.30	0.37	3.87	1.43
6	26.28 ± 7.02	0.43	3.77	1.64
10	28.30 ± 8.93	0.46	4.46	2.04
15	42.68 ± 15.05	0.96	3.88	3.71
21	65.36 ± 22.85	1.29	3.69	4.75
